# Early stages of growth of gold layers sputter deposited on glass and silicon substrates

**DOI:** 10.1186/1556-276X-7-241

**Published:** 2012-05-06

**Authors:** Petr Malinský, Petr Slepička, Vladimír Hnatowicz, Václav Švorčík

**Affiliations:** 1 Nuclear Physics Institute, Rez, 250 68, Czech Republic; 2 Department of Solid State Engineering, Institute of Chemical Technology in Prague, Prague, 166 28, Czech Republic

**Keywords:** sputtering, gold layer, glass, silicon, RBS

## Abstract

Extremely thin gold layers were sputter deposited on glass and silicon substrates, and their thickness and morphology were studied by Rutherford backscattering (RBS) and atomic force microscopy (AFM) methods. The deposited layers change from discontinuous to continuous ones for longer deposition times. While the deposition rate on the silicon substrate is constant, nearly independent on the layer thickness, the rate on the glass substrate increases with increasing layer thickness. The observed dependence can be explained by a simple kinetic model, taking into account different sticking probabilities of gold atoms on a bare glass substrate and regions with gold coverage. Detailed analysis of the shape of the RBS gold signal shows that in the initial stages of the deposition, the gold layers on the glass substrate consist of gold islands with significantly different thicknesses. These findings were confirmed by AFM measurements, too. Gold coverage of the silicon substrate is rather homogeneous, consisting of tiny gold grains, but a pronounced worm-like structure is formed for the layer thickness at electrical continuity threshold. On the glass substrate, the gold clusters of different sizes are clearly observed. For later deposition stages, a clear tendency of the gold atoms to aggregate into larger clusters of approximately the same size is observed. At later deposition stages, gold clusters of up to 100 nm in diameter are formed.

## Background

Coating of substrates with thin metal films is of great importance for contemporary technologies (optical coatings, corrosion protection, semiconductor devices). Different methods, such as sputtering, pulsed laser deposition, vacuum evaporation, vapor phase techniques, or molecular beam epitaxy, are used for thin film creation. Growing rate and resulting morphology of the thin metal films depends on the deposition technique used and several other factors including the properties of substrate, adsorbed atoms, and their interaction strength with the substrate surface. Initial phase of the film growth and its morphology is a persisting problem in the physics of thin films and in a wide range of technological applications, too. Three basic mechanisms of the film growth have been described and observed [1].

Experimental examination of the film morphology in the initial phases of growth is not easy despite the wide spectrum of diagnostic techniques available for this purpose, including AES, LEED, RHEED, XPS, atomic force microscopy (AFM), SEM, Rutherford backscattering (RBS), and TEM. The properties of the metal films deposited by various techniques on different substrates have been the subject of many studies: experimental and theoretical (see e.g. [2-6]). Gold is often used in these studies because of its inert character and the interesting physicochemical properties of gold nanoparticles.

In this work, standard RBS and AFM methods are used for the study of growth and morphology of very thin gold layers prepared by sputtering on two different substrates: ‘common’ glass and monocrystalline Si. The study is a continuation of our previous works in the field.

## Methods

### Substrate and Au deposition

The gold films were deposited onto two substrates: standard Si (100) n-type wafer (5-cm diameter) and glass (25 × 25 mm^2^, 0.1-mm thick, Marienfeld, Lauda-Königshofen, Germany). The native SiO_2_ was present on the Si substrate. The substrates were cleaned with methanol; the drying of samples was performed with nitrogen flow. Deposition was performed by diode sputtering from a 99.99 % Au target (supplied by Goodfellow Ltd, Friedberg, Germany) using a Bal-Tec SCD 050 device (BalTec Maschinenbau AG, Pfäffikon, Switzerland). The sputtering conditions were a discharge power of 7.5 W, a total pressure of about 5 Pa of argon (99.995 % purity), and the electrodes of 48 cm^2^ in area at a distance of 50 mm. The deposition was performed at room temperature, and the deposition times from 5 to 150 s were chosen to create the layers from discontinuous to continuous ones. The deposition onto both substrates was performed simultaneously.

### Diagnostic techniques

RBS analyses were performed on a Tandetron 4130MC accelerator in the Nuclear Physics Institute in Rez using 1.75-MeV ^4^He ions. The measurements were performed in IBM geometry with an incident angle of 0° and a laboratory scattering angle of 170°. Scattered particles were registered with a surface barrier detector connected to a standard spectrometric chain and acquisition system. The typical energy resolution of the spectrometer was FWHM = 15 keV. The ion flux was measured using a monitor with a rotating target situated in front of the sample. Sufficiently long measuring times were chosen to reduce typical statistical errors in gold signal to about 1 %. The RBS spectra were evaluated using a SIMNRA code [7].

Surface morphology and roughness of the pristine and gold-deposited Si and glass samples were examined by AFM technique using a VEECO CP II device in tapping mode (Bruker Corporation, Santa Barbara, CA, USA). A Si probe RTESPA-CP with a spring constant of 20 to 80 N m^−1^ was used. The mean roughness value (*R*_a_) represents the arithmetic average of the deviations from the center plane of the sample. AFM technique combined with standard scratch method was also used for the determination of the gold layer thickness [8].

## Results and discussion

**Figure 1:**
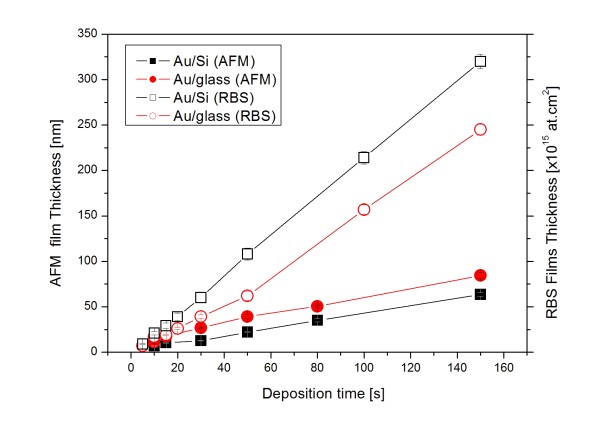
**Layer thickness.** Thickness of the gold layer deposited on the glass and Si substrates as a function of the deposition time determined by RBS and AFM methods. For RBS data, measuring statistical errors of about 1 % are not shown. Note that the thicknesses are given in atom area density and in nanometers for RBS and AFM data, respectively. The area density can be converted into nanometers by multiplying it with an approximate factor of 0.1695.

**Figure 2:**
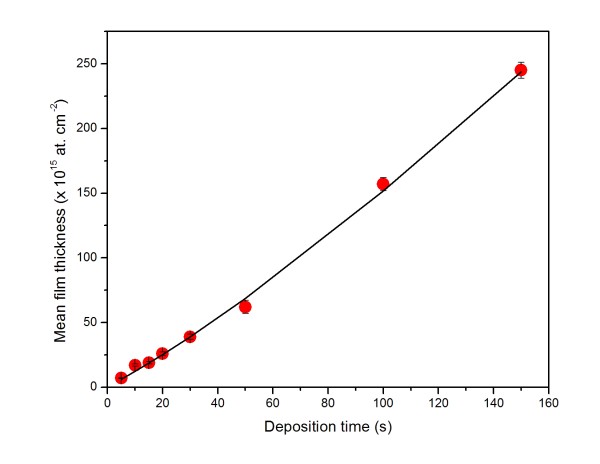
AFM images of pristine glass (glass) and glass sputtered with gold for 30 s (glass/Au 30) and for 150 s (glass/Au 150). *R*_a_ represents average surface roughness in nanometers; AFM images in both two-dimensional (2D) and three-dimensional (3D) views are shown.

The thicknesses of the gold layers, determined from RBS spectra and from AFM measurement for both Si and glass substrates, are shown in Figure 1 as a function of the deposition time. The thicknesses are presented in natural units of both methods, i.e., in atom area density (RBS) and in nanometers (AFM). Since the density of the deposited gold layer is not known and may differ from that of bulk gold significantly, the area density can not be converted into nanometers correctly. Nevertheless, using deliberately bulk gold density (19.3 g cm^−3^), a factor of 0.1695 is obtained for conversion of the area density (in 10^15^ at.cm^−2^) into nanometer units. As could be expected, the layer thickness is a monotonously increasing function of the deposition time. However, one can see that the data obtained by RBS and AFM methods differ in some respects, the differences being obviously due to different principles of both methods. While from RBS spectra only the mean layer thickness, averaged over the beam spot (usually several square millimeters in area), is determined, the data obtained by AFM combined with scratch technique are absolute but strongly affected by the local nanostructure of the gold layer [8]. In contrast to RBS data, the layer thicknesses on the glass substrate obtained with AFM are higher. The difference is due to the presence of large clusters in the layers formed on the glass substrate (see Figure 2).

One can see from the RBS data that there is a great difference in the deposition rates on both substrates, the deposition on the Si substrate being faster. While the deposition on Si proceeds with nearly constant deposition rate, in the case of the glass substrate, the deposition in the initial stages of the layer growth proceeds with significantly lower deposition rate. It should be noted that according to our previous studies [5,6], the gold layers deposited for the shortest deposition times are discontinuous, i.e., composed of separated gold islands. Observed evolution of the deposition rate on the glass substrate may be due to different probabilities of gold atom capture on the bare glass substrate and already created gold islands. To describe the observed evolution of the mean layer thickness *n*(t) (in at.cm^−2^) with the sputtering time *t*, a simple kinetic model is suggested, supposing that there are *N* sites per unit area on the substrate surface which may be occupied by one or more incoming gold atoms. Further, it is supposed that the probability of the capture of gold atoms on the already occupied site is 100 % and that on the still empty site is reduced by a factor *f* < 1. According to the Poisson statistics, the instantaneous probability that a site is still empty is $\text{exp}\left(\frac{-n\left(\text{t}\right)}{N}\right)$, and the probability that it is already occupied by one or more gold atom is $1-\text{exp}\left(\frac{-n\left(\text{t}\right)}{N}\right)$. Under these conditions, the growth of the mean layer thickness is described by the kinetic equation:

(1) $$\frac{dn\left(t\right)}{dt}=j\left(\text{fexp}\left(\frac{-n\left(t\right)}{N}\right)+1-\exp\left(\frac{-n\left(t\right)}{N}\right)\right)=j.\left(1-\left(1-f\right)\exp\left(\frac{-n\left(t\right)}{N}\right)\right)$$

where *j* is the number of incoming gold atoms in cm^−2^ s^−1^ units. One can see that with the increasing thickness of the layer, the deposition rate increases as expected. The equation can easily be solved and obtained for the mean layer thickness:

(2) $$n\left(t\right)=j.t+N.\ln\left(1-\left(1-f\right)\left(1-\exp(\frac{-j.t}{N}\right)\right)$$

**Figure 3:**
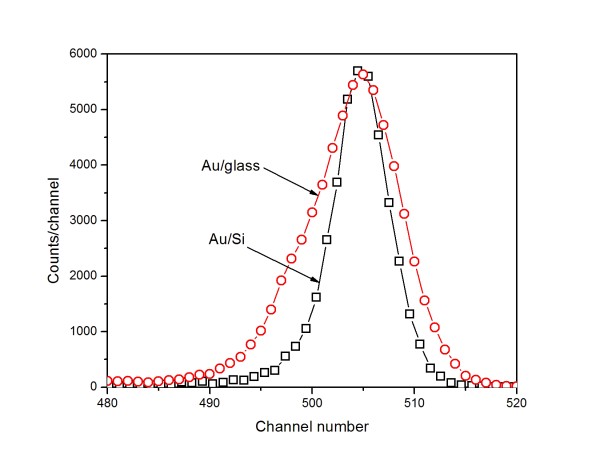
**Least squares fit.** The NLSQ fit (solid line) of the measured dependence of the mean thickness of the gold layer deposited on the glass substrate (points).

To determine deposition rates, the measured dependence of the mean thickness of the gold layer on the deposition time was fitted with this formula, using standard nonlinear least squares technique (NLSQ), *jN*, and *f* being the adjustable parameters. The quality of the fit is illustrated by Figure 3 with the data on the glass substrate. It is found that Equation 2 describes well the data obtained on both Si and glass substrates. The resulting values of the fitted parameters are summarized in Table 1. The values of the incoming flux of gold atoms, *j*, for both substrates are close with each other, as could be expected. A rather great difference in the number of sites, *N*, is obviously due to different surface properties of both substrates, which in turn may affect the behavior of the deposited gold atoms. One can see that the probability of the capture of gold atoms on bare Si, 92 %, is close to that on already existing gold islands (deliberately chosen as 100 %). For the glass substrate, this probability is only 59 %. It should, however, be noted that the above model simplifies greatly the real situation. In the deposition process, several other processes, such as surface diffusion, aggregation, re-emission of gold atoms, etc., may play a significant role [1].

**Table 1:** Parameters of Equation 2

**Substrate**	***j***	***N***	***f***
	**(10**^**15**^ **cm**^**−2**^ **s**^**−1**^**)**	**(10**^**15**^ **cm**^**−2**^**)**	
Glass	2.00 ± 0.02	59.70 ± 3.0	0.59 ± 0.02
Si	2.16 ± 0.02	28.9 ± 7.5	0.92 ±0.02

Obtained by NLSQ fitting of measured dependences of the mean layer thickness on the deposition time (see Figure 1).

**Figure 4:**
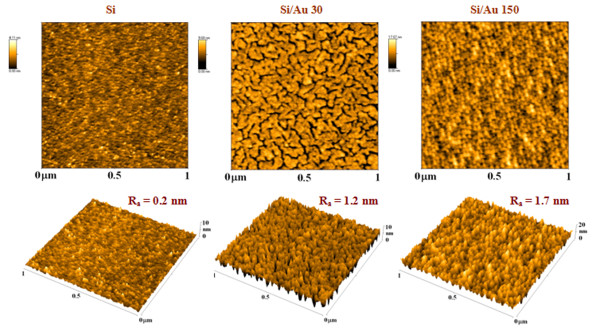
**Normalized part of RBS spectrum.** The part of RBS spectra (based on RBS measurement) on the samples with gold layers sputtered for 5 s onto Si and glass substrates. For the sake of comparison, the spectra were normalized to the same maximum amplitude.

It is well known that the morphology of the nano-sized objects affects the form of the RBS spectra from which a useful information on the object structure may be obtained (see e.g. [9,10]). In Figure 4, the relevant parts of the RBS spectra with gold signal from the samples with the gold layer sputtered for 5 s onto the glass and Si substrates are compared. While the signal from the gold layer on the Si substrate has been expected to be nearly of Gaussian shape with the width close to the spectrometer energy resolution, the signal from the gold layer deposited on the glass substrate is by about 50 % broader and exhibits significant left-hand asymmetry. A similar but not so strong difference is observed on the samples sputtered for 10 s, and for longer sputtering times, the difference disappears. In Table 2, the relevant information for the gold layers sputtered from 5 to 20 s is summarized. The broader and asymmetric signal observed on the samples sputtered on the glass substrate indicates that the thin, discontinuous layers are probably composed of gold islands, the thickness of which varies significantly. In the later phases of deposition, the homogeneity of the deposited layer improves rapidly.

**Table 2:** **Widths of gold signal calculated as central second moment of measured distribution (see Figure**4**)**

**Sputtering time** **(s)**	**Glass substrate**		**Si substrate**	
	***σ***	***S***	***σ***	***S***
	**(keV)**	**(10**^**15**^**at.cm**^**−2**^**)**	**(keV)**	**(10**^**15**^**at.cm**^**−2**^**)**
5	4.6	53	2.9	34
10	4.8	55	3.0	35
15	3.2	37	3.2	37
20	3.3	38	3.2	37

Typical statistical error is below 10 %.

**Figure 5:**
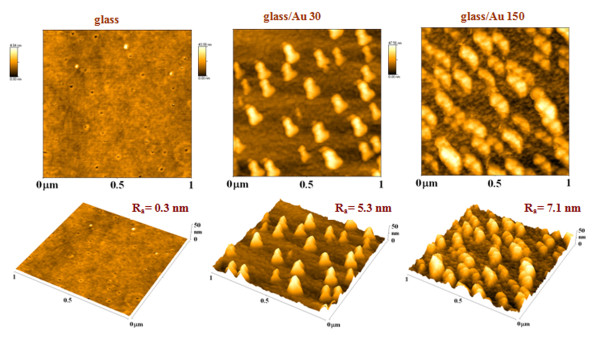
**AFM images of pristine Si and gold-coated Si.** AFM images of pristine Si (Si) and Si sputtered with gold for 30 s (Si/Au 30) and for 150 s (Si/Au 150). *R*_a_ represents the average surface roughness in nanometers; AFM images in both 2D and 3D views are shown.

**Figure 6:**
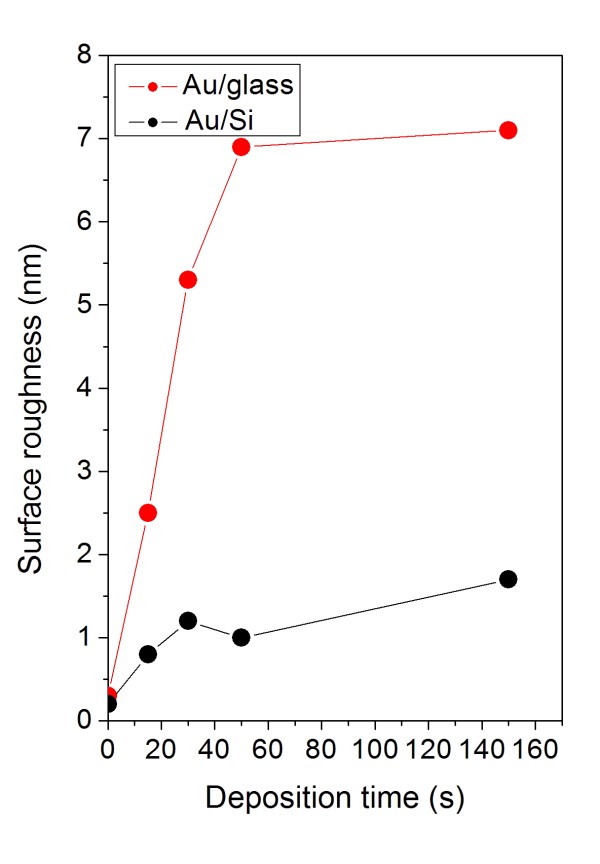
**Surface roughness.** Dependence of *R*_a_ (in nanometers) of pristine and Au-covered glass and Si substrates on deposition time.

Surface morphologies of the bare glass and silicon substrates and the substrates coated with gold were examined by AFM method, and the typical AFM images are shown in Figures 2 and 5. Bare Si surface exhibits flat morphology with low surface roughness not exceeding 0.2 nm. The gold layers, deposited on the silicon substrate, are rather homogenous consisting of small gold particles. With increasing deposition time, the layer morphology changes only slightly and the *R*_a_ increases slowly (Figure 6). In the layer deposited for 30 s, a worm-like structure is formed (see Figure 5). This type of metal-substrate structure was also observed in our previous experiments performed on polymer (PET) and glass substrates [11-13]. According to our previous studies, this stage of gold layer growth coincides with the transition phase from the electrically discontinuous to continuous gold layer [13,14]. At later deposition stages, a globular structure of the gold layer is observed. It should be noted that in the present experiment, gold deposition was accomplished with twice as high sputtering current compared to our previous experiments [13].

Pristine glass surface exhibits very low surface roughness compared to the pristine Si surface (see Figures 2, 5, and 6). A rather complex surface morphology is observed on the gold-covered glass. The presence of isolated gold grains of various sizes is observed for all deposition times. With increasing deposition time, the mean size and the density of these grains increase, but the initial size differences (observed at deposition times of 5 and 15 s) are gradually smeared out. The later effect is reflected in the evolution of the *R*_a_ which increases rapidly in initial deposition stages and achieves saturation for later ones (Figure 6). The gold grains with diameters of up to 100 nm are formed in the later deposition stages. With increasing deposition time, the gold layer becomes electrically conductive. These findings are in agreement with above-mentioned RBS results, namely with the evolution of the width and asymmetry of the RBS gold signal (see also Table 2). The appearance of the gold grains and their growth may be due to the above-mentioned preferential capture of the incoming gold atoms on the already existing gold islands or to surface diffusion of deposited gold atoms and their aggregation into larger grains.

## Conclusions

Thickness of gold layers sputtered onto silicon and glass substrates as a function of the deposition time was measured by standard RBS method and AFM combined with scratch technique. The layer thickness is a monotonously increasing function of the deposition time, as could be expected. The deposition rate on the silicon substrate determined by the RBS practically does not depend on the instantaneous layer thickness. For the glass substrate, however, the deposition rate is low for short deposition times and discontinuous gold coverage, and with increasing deposition time and more homogenous coverage, it increases to a value comparable with that found on the silicon substrate. The observed rate dependence can be explained by different sticking probabilities of gold atoms on the bare glass substrate and on regions with gold coverage. The results on the layer thicknesses obtained by AFM method differ from those by RBS significantly, the differences being due to different principles of both methods, namely, in contrast to the RBS results, the AFM method gives higher layer thicknesses on the glass substrate than on the silicon substrate. The AFM images taken on bare substrates and those coated with gold for different deposition times show great differences in the morphology of the gold layers deposited on both substrates. On the silicon substrate, rather homogenous gold layers are observed, the surface morphology of which has only little changes with increasing sputtering time. At the deposition stage roughly corresponding to the outbreak of layer electrical conductivity, a worm-like structure in the gold layer is formed, and at later deposition stages, a globular layer structure is observed. On the glass substrate, however, the presence of isolated gold grains of various sizes is observed for short deposition times. With increasing deposition time, the mean size and the density of these grains increase, but the initial differences in the grain size are gradually smeared out. The AFM results are in good agreement with the results of RBS measurements, especially with detailed analysis of the form of the gold RBS signal, indicating significant heterogeneity of the gold layers deposited for short times on the glass substrate.

Present results, together with our previous ones, contribute to a better understanding of initial stages of the formation of metal layers on different substrates. The results can be of interest for specialists developing metamaterials, photonic crystal structures, and nanostructured materials with enhanced biocompatibility.

## Competing interests

The authors declare that they have no competing interests.

## Authors’ contributions

PM carried out the sample preparation and RBS analysis and designed the study. PS analyzed the surface morphology and evaluated the surface roughness and thickness. VH carried out the mathematical fitting and participated on the RBS analysis. VH and VŠ conceived the study and participated in its coordination. All authors read and approved the final manuscript.
